# Possible A2E Mutagenic Effects on RPE Mitochondrial DNA from Innovative RNA-Seq Bioinformatics Pipeline

**DOI:** 10.3390/antiox9111158

**Published:** 2020-11-20

**Authors:** Luigi Donato, Concetta Scimone, Simona Alibrandi, Alessandro Pitruzzella, Federica Scalia, Rosalia D’Angelo, Antonina Sidoti

**Affiliations:** 1 Department of Biomedical and Dental Sciences and Morphofunctional Imaging, Division of Medical Biotechnologies and Preventive Medicine, University of Messina, 98125 Messina, Italy; ldonato@unime.it (L.D.); salibrandi@unime.it (S.A.); rdangelo@unime.it (R.D.); asidoti@unime.it (A.S.); 2 Department of Biomolecular strategies, genetics and avant-garde therapies, I.E.ME.S.T., 90139 Palermo, Italy; 3 Department of Chemical, Biological, Pharmaceutical and Environmental Sciences, University of Messina, 98125 Messina, Italy; 4 Department of Biomedicine Neuroscience and Advanced Diagnostics, University of Palermo, 90127 Palermo, Italy; alessandro.pitruzzella@unipa.it (A.P.); scalia.fede@gmail.com (F.S.)

**Keywords:** mitochondria, mtDNA, RNA-Seq, retinal degenerations, VUS

## Abstract

Mitochondria are subject to continuous oxidative stress stimuli that, over time, can impair their genome and lead to several pathologies, like retinal degenerations. Our main purpose was the identification of mtDNA variants that might be induced by intense oxidative stress determined by *N*-retinylidene-*N*-retinylethanolamine (A2E), together with molecular pathways involving the genes carrying them, possibly linked to retinal degeneration. We performed a variant analysis comparison between transcriptome profiles of human retinal pigment epithelial (RPE) cells exposed to A2E and untreated ones, hypothesizing that it might act as a mutagenic compound towards mtDNA. To optimize analysis, we proposed an integrated approach that foresaw the complementary use of the most recent algorithms applied to mtDNA data, characterized by a mixed output coming from several tools and databases. An increased number of variants emerged following treatment. Variants mainly occurred within mtDNA coding sequences, corresponding with either the polypeptide-encoding genes or the RNA. Time-dependent impairments foresaw the involvement of all oxidative phosphorylation complexes, suggesting a serious damage to adenosine triphosphate (ATP) biosynthesis, that can result in cell death. The obtained results could be incorporated into clinical diagnostic settings, as they are hypothesized to modulate the phenotypic expression of mtDNA pathogenic variants, drastically improving the field of precision molecular medicine.

## 1. Introduction

### 1.1. Mitochondria Represent the Most Intriguing Organelle of Eucaryotic Cell

Mitochondria represent one of the most crucial and interesting organelles of eukaryotic cells. The major role of mitochondria consists of production of cellular adenosine triphosphate (ATP) and the establishment of membrane potential by oxidative phosphorylation [1]. Such activities can be realized thanks to the involvement of a huge number of proteins, most of which are encoded by the nuclear genome and then translocated to mitochondria [2]. However, the most exciting aspect of mitochondria resides in the uniqueness of its own genome (mtDNA), a double-stranded circular molecule of about 16,600 nt encoding for only thirteen polypeptides of the oxidative phosphorylation complex (OXPHOS), along with twenty-two tRNAs and two rRNAs (12S and 16S). The most polymorphic site of mtDNA is the non-coding portion situated within a 1 kb noncoding region (NCR), which can regulate both transcription and translation [3].

### 1.2. mtDNA Damage is Linked to Heterogeneous Clinical Phenotypes

Today, it is well known that a protracted accumulation of lower levels of mtDNA damage and mtDNA copy reduction could be linked to the etiopathogenesis of neurodegenerative and metabolic age-related diseases [4]. Mitochondrial damages, showing an incidence of about 1:4300, primarily affect oxidative phosphorylation but, due to several mitochondrial proteins encoded by the nuclear genome, the derived clinical phenotypes are significantly heterogeneous [5]. Despite the actual number of about 15,000 variants reported in the constantly updated MITOMAP human mitochondrial genome database [6], only a few hundred are confirmed as disease causing. These mutations are at the basis of a wide spectrum of maternally inherited diseases, characterized by high heterogeneity of both pathological phenotype and penetrance, primarily deriving from shifts and differences in the mutant load, due to stochastic segregation of mtDNA during cellular divisions. Consequently, the mutation load could range from 100% mutant load (homoplasmy) to the coexistence of both mutant and wildtype molecules (heteroplasmy), also varying across different tissues and organs. An increase in the level of heteroplasmy corresponds to a decrease in energy production to the minimum threshold needed for cell physiological homeostasis, leading to the onset of symptoms [7].

### 1.3. Next Generation Sequencing Techniques could Reveal a Relevant Role of mtDNA Impairments in Retinal Degenerations

Several reports highlighted mtDNA damage as an important contributing factor in retinal degeneration-related pathologies [8,9]. It was stated that mtDNA is more disposed to damage than nuclear DNA, and it seems that retinal mtDNA damage preferentially occurs in the macula rather than in the periphery [10]. Another challenge is represented by mutagenesis of mtDNA related to the efficacy of DNA repair enzymes, encoded by the nuclear genome but imported into the mitochondria to exert their function [11]. Therefore, DNA damage response in mitochondria (mtDDR) in retinal diseases presents many unknown molecular aspects that should be unveiled. Today, the advances in next generation sequencing (NGS) techniques has permitted to perform reliable analysis of mtDNA, improving sample output and sensitivity of variant detection [12], even if several limits remain. Main challenge that will be faced working with mtDNA massive parallel sequencing regard variants unrelated to exhibited phenotype, detection and interpretation of low heteroplasmy and homoplasmy levels and identification of variants of unknown significance (VUS) [13].

### 1.4. Evaluating mtDNA Damage by Mitochondrial Transcript Analysis in Photo-Induced Oxidative Stressed RPE Cells could Shed Light on the Role of Mitochondria in Retinal Dystrophies

To better understand how high ROS levels induced by oxidative stress can damage mtDNA, influencing retinal degeneration onset and progress, we performed a variant analysis comparison from transcriptome profiles of human RPE cells exposed to the oxidant agent *N*-retinylidene-*N*-retinylethanolamine (A2E) and untreated ones. The RPE cells are highly susceptible to oxidative/nitrosative stress, because they are frequently exposed to blue light and reside in an environment with high oxygen tension [14]. The most reliable model depicting cellular causes of this condition regards the blue light induced mitochondrial fusion/fission imbalance towards mitochondrial fragmentation. This phenomenon correlated with the dysregulation of mitochondria-shaping and mitochondria dynamics-related protein levels, together with up-regulation of mitochondrial mitotic proteins and down-regulation of fusion proteins [15]. Moreover, blue light could destroy mitochondrial calcium homeostasis, impairing the transmembrane potential (MPP) and increasing mitochondrial membrane permeability [16]. The first stages of RPE degeneration usually imply the accumulation of metabolic waste between choroidal and RPE layers, called drusen. Drusen consist of mixtures of high photo-sensitive lipofuscin, involved in the first photo-sensitive reactions, caused by the generation of singlet oxygen and superoxide anion. A2E is one of the most abundant components of drusen, and a direct transmittal of light to the retina by drusen determines its cleavage at the pyridinium ring, inducing oxidative stress [17,18,19]. Thus, the main purpose of our study was the identification of mtDNA variants that might be induced by intense oxidative stress, together with their possible related molecular pathways most likely linked to retinal degeneration onset and/or progression. We tried to reach this objective using an innovative pipeline, starting from RNA-Seq data and focusing on mitochondrial transcripts.

## 2. Materials and Methods

### 2.1. Cell Culture Samples

Human Retinal Pigment Epithelial Cells (H-RPE, Clonetics™, Lonza, Walkersville, MD, USA) were cultivated and then grown for 24 h until confluence, as previously described [20]. Successively, a group of cells was treated with A2E 20 μM for 3 h and 6 h before rinsing with medium, while a control group was incubated without the oxidant compound. Lastly, confluent cultures were transferred to PBS-CMG and then subjected to blue light delivered by a tungsten halogen source (470 ± 20 nm; 0.4 mW/mm^2^) for 30 min, in order to induce phototoxicity of A2E, and incubated at 37 °C for 24 h. Each sample of cells foresaw three biological replicates.

### 2.2. MTT Assay

The cell viability was evaluated by the mitochondrial-dependent reduction of methylthiazolyldiphenyl-tetrazolium bromide (MTT) (Sigma-Aldrich, St. Louis, MO, USA) to formazan insoluble crystals, following an already defined protocol [20]. Ultimately, a Dynatech microplate reader allowed us to estimate absorbance at 570 nm, and the results were expressed as a percentage of viable cells compared with control conditions in the absence of A2E. Multiple *t*-tests were performed for statistical comparisons (*p* < 0.05), considering three independent experiments, each one characterized by three replicates.

### 2.3. Whole RNA Extraction and RNA-Seq Profiling

Total RNA was isolated, checked for degradation and contamination, and quantified as previously reported [20]. The RNA-seq samples were classified in 3 factor groups, made of human RPE cells before A2E treatment and at the different time points of 3 h and 6 h, respectively. Each group was biologically replicated three times, for a total of 9 samples. Both 3 h and 6 h time points were chosen considering experiments previously realized by our research group (unpublished data), confirmed by outcomes derived from MTT assay in this work. Such results highlighted that in wider time intervals the death rate of oxidative stressed cells might be so high as to invalidate the following data analysis. Libraries were generated using 1 µg of total RNA by the TruSeq Stranded Total RNA Sample Prep Kit with Ribo-Zero H/M/R (Illumina, San Diego, CA, USA), following manufacturer’s protocols. The concluding step foresaw the sequencing of the libraries on an HiSeq 2500 Sequencer (Illumina, San Diego, CA, USA), using the HiSeq SBS Kit v4 (Illumina, San Diego, CA, USA).

### 2.4. Mitogenome Assembly/Mapping

**Figure 1:**
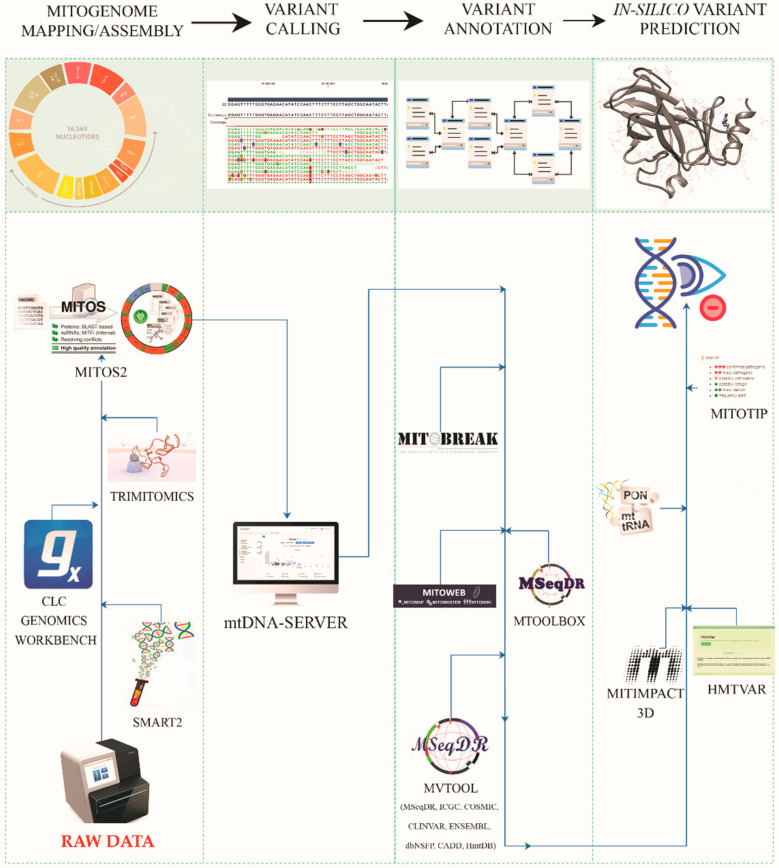
Proposed pipeline of mtDNA variant analysis. Figure shows applied workflow of mtDNA variant analysis, highlighting the single macro-steps with specific tools and explored databases.

To perform an accurate and reliable analysis of mtDNA variants produced by transcriptome experiments, obtaining a higher quality output, we propose an integrated approach that foresees the complementary use of the most recent algorithms applied to mtDNA data, adapting them to a mitochondrial transcript source of data. A schematic workflow of the entire pipeline is reported in Figure 1.

**Figure 2:**
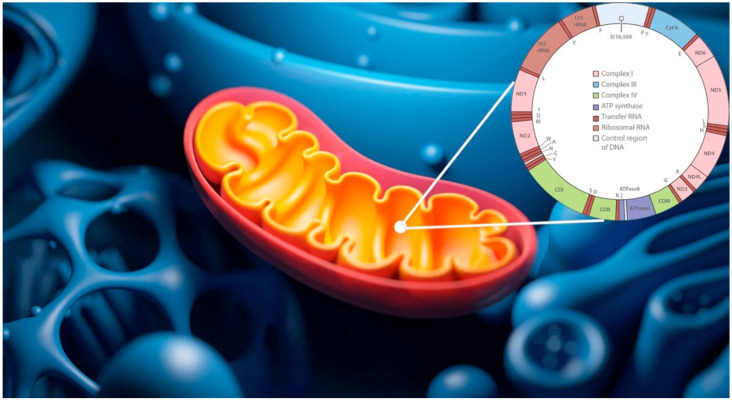
Revised Cambridge Reference Sequence (rCRS) reference mtDNA sequence. Figure shows main genomic features of Revised Cambridge Reference Sequence (rCRS), available as sequence number NC_012920 in the GenBank RefSeq database.

Generated raw sequences were filtered for low-quality reads (average per base Phred score < 30) and adaptor sequences. The quality of the analyzed data was assessed by FastQC (v.0.11.9) [21] and QualiMap (v.2.2.1) [22], while trimming was performed by Trimmomatic (v.0.39). Filtered data was, then, assembled/aligned by CLC Genomics Workbench v.20.0.4 [23], Multi-Sample Statistical Mitogenome Assembly with Repeats (SMART2) [24] and an adapted version of TRIMITOMICS pipeline [25]. Previously, data analyses were realized using the Revised Cambridge Reference Sequence (rCRS), available as sequence number NC_012920 (formerly AC_000021.2) in GenBank’s RefSeq database. This specific rCRS, made of 16,569 bp, is the most commonly used standard comparison sequence for human mtDNA research (Figure 2). It is a single reference individual from haplogroup H2a2 and has been used as a standard for reporting variants for over 30 years.

Mapping analysis with the CLC platform was conducted using the following settings: quality trim limit = 0.01, ambiguity trim maximum value = 2. The map to annotated reference was as follows: mismatch cost = 2, insertion and deletion costs = 3, minimum length fraction and minimum similarity fraction = 0.8, maximum number of hits for a read = 10, strand-specific = both.

SMART2, the most recent pipeline able to assemble de novo and annotate complete a circular mitochondrial genome sequence from NGS sequencing data, was set as follows: automatic selection of number of read pairs per bootstrap, with doubling strategy starting with 100 k; number of bootstrap samples = 1; minimum seed kmer coverage = 20; coverage-based filtering method = intersection; kmer size = 31; number of threads = 16; genetic code = 02-vertebrate.

The most powerful pipeline we used was TRIMITOMICS, particularly interesting for the assembly of mitochondrial gene cassettes and whole coding sequences from RNA-Seq reads. It is based on free algorithms used stepwise, depending on the success of mitogenome assembly in the preceding step. The first step foresaw the use of NOVOPlasty v.3.8.2 organelle assembler, set as follows: Genome Range = 1–16,569; k-mer = 31; max memory = 16; extend seed directly = no; variance detection = no. If a complete or partial mtDNA was not obtained, the RNA-Seq reads were firstly mapped to their respective reference genome with Bowtie2 v.2.4.1 algorithm, using standard settings, and then assembled with Trinity v.2.10.0, following a genome guided approach with default presets, with the exception of the parameter “max intron length = 10,000”. If none of the previously cited methods successfully produced mitogenome, the complete transcriptome would have been assembled by Velvet v.1.2.10, considering a range of kmer sizes (31, 51, 71). Mitochondrial contigs were then extracted from de novo generated transcriptome assemblies by BlastN, using the reference mtDNA. If the complete genome was not retrieved by any of the described approaches, the obtained partial results were joined or put together as a meta-assembly with MAFFT v.7.464 to improve the output.

### 2.5. Variant Detection by mtDNA-Server

Once obtained, assembled mtDNAs were firstly validated and then analyzed for variant calling by mtDNA-Server, a highly scalable Hadoop-based server for mtDNA NGS data processing [26]. HadoopBAM split input into several chunks and, for each one, only reads with Phred score > 20 and length > 25 were maintained, while reads marked as duplicates were filtered out. Afterwards, all passed bases for each site were counted per strand (A, C, G, T, N – unknown - and d – deletion-). Heteroplasmy detection was performed following different approaches: initially, sites presenting coverage < 10 bases per strand and mitochondrial hotspots around 309, 315 and 3107 were filtered out, according to reference sequence. For survived sites with an allele coverage of 3 bp per strand and a variant allele frequency (VAF) ≥ 1% (strand independent), a machine learning model was applied, considering sequencing errors per base in each strand. Then, all sites showing a log likelihood ratio (LLR) ≥ 5 were marked as heteroplasmic sites. Moreover, the Wilson and the Agresti-Coull confidence intervals were calculated for heteroplasmic variants, and the assigned heteroplasmy level was a weighted mean of heteroplasmy of both strands. An important feature of the mtDNA-Server regards the intra-sample contamination check, based on current phylogeny to avoid erroneous interpretations and conclusions. In the case of contaminations caused by different mtDNA sequences, the two VAF-based profiles generated by the mtDNA-Server (VAF < 50% for the minor, VAF > 50% for the major) led to different valid haplogroups.

### 2.6. Variant Annotation and Prioritization

This critical step was performed following several practical rules: (1) particular attention to low heteroplasmy levels, (2) evaluation of variant frequency both in the general population and in particular haplogroups, (3) analysis of additional data supporting the modulation of clinical penetrance, such as mitochondrial haplogroup, 4) consideration of inter-species nucleotide and/or amino acid conservation.

The most complete tool we used to realize these purposes was the MSeqDR mtDNA Variant Tool set (mvTool), built upon the MSeqDR infrastructure [27]. It supports all mtDNA nomenclatures, converts variants to standard rCRS- and HGVS-based nomenclatures, and annotates novel mtDNA variants [28]. For already annotated mitogenome variants, mvTool extracted and provided updated population data and pathogenetic classifications from MSeqDR Consortium members [29], the Human Mitochondrial Database (HmtDB) [30], dbNSFP [31], ClinVar [32], Mitomap [33], the 1000 Genomes Project data and GeneDx [34], with resources coming from around 50,000 germline mtDNAs. For variants that had not been annotated before, mvTool conducted new predictions by calling Ensembl Variant Effect Predictor (VEP) [35] and stored its genomic annotations in an internal database that mvTool searched first. Additionally, for input including all mitogenome variants of a given sample, exact mtDNA haplogroup assignment was obtained by PhyMer sub-tool.

Furthermore, we applied a de novo annotation pipeline to evaluate and improve existing annotations, trying to reduce inconsistencies and errors, such as missing gene annotations, missing or incorrect information of the reading direction (strand), mistaken identity of tRNAs, erroneous gene designations and inconsistencies in gene names. To reach this purpose, the MITOchondrial genome annotation Server 2 (MITOS2) was used [36]. It exploits a novel strategy based on aggregating BLAST searches with previously annotated protein sequences to detect protein coding genes, tRNAs and rRNA. Each structured RNA is then annotated using specific covariance models.

Nevertheless, to simplify variant frequency interpretation of a general population, frequently challenging due to mtDNA genetics feature (heteroplasmy level, incomplete penetrance, influence of mitochondrial haplogroup background), we enriched our analytic pipeline with tools and databases focusing on haplogroup classification. The already cited Mitomap advised whether a variant was identified at >1% in at least one of the macro-lineages or over 10% in the major haplogroups for tRNA variants. Results from this step of prioritization were then corroborated by data coming from MToolBox, which applied a computational strategy to realign already assembled mitochondrial genomes to detect InDels and to assess the heteroplasmic fraction (HF) of each variant allele with the related confidence interval (CI), before haplogroup assignment and variant prioritization [37]. This latter step was realized by aligning each sample-specific reconstructed contig against the related macro-haplogroup-specific consensus sequence. This process could detect private variants through a prioritization process, justifying further clinical investigation. Prioritization also considered the pathogenicity of each mutated allele, computed with different algorithms, and the nucleotide variability of each variant site, while the amino acid variability was considered only if the variant site was codogenic.

Finally, to complete the variant data, we retrieved records from the MitoBreak database, focusing on mtDNA rearrangements following breakpoints from linear mtDNAs, circular deleted mtDNAs (deletions) and circular partially duplicated mtDNAs (duplications) [38].

### 2.7. In Silico Predictions and Variant Consequences

Currently, massive mtDNA screening by NGS is showing a relevant number of novel variants of unknown significance (VUS), whose clinical interpretation is more complicated than nuclear VUS, due to the already cited challenging mitogenome characteristics and due to limited guidelines for mtDNA compared to those provided for nuclear VUS.

Thus, to obtain reliable results, we performed a combined approach made of complementary in-silico prediction tools. These tools evaluated the variant functional impact by algorithms based on interspecies sequence conservation and/or structure analysis.

The most complete and continuously updated tool we used in this step was MitImpact 3D v.3.0.2, a collection of pre-computed pathogenicity prediction scores for all possible nucleotide changes that determine non-synonymous substitution, in human mitochondrial protein coding genes [39]. We evaluated the following MitImpact 3D missense pathogenicity predictors and machine learning based approach meta-predictors: SIFT (v.5.0.3) [40], PolyPhen2 (v.2.2.2) [41], MutationAssessor (v.2.0) [42], PANTHER [43], FatHmm (v.2.2, “weighted” and “unweighted” setting) [44], PROVEAN (v.1.3) [45], CADD (v.1.2) [46], EFIN [47], SNAP [48], PhD-SNP [49], MutationTaster v.2 [50], COVEC (v.0.4) [51], SNPdryad [52], DEOGEN2 [53], Mitoclass.1 [54], CAROL [55], Meta-SNP [56], Condel [57], APOGEE (v.1.0) [58], ClinVar, dbSNP (v.151) [59], PhyloP and PhastCons evolutionary conservation indices (UCSC Gene Tables, group: Comparative Genomics; track: Conservation; tables: phyloP100wayAll and PhastCons100way) [60], SiteVar human mtDNA site-specific variability [61], MISTIC Mutual Information scores [62], COSMIC somatic variants (ver. 87) [63], TransFIC [64], CHASM [65]. Additionally, the tool permitted the evaluation of compensated pathogenic deviations (CPDs), amino acid substitutions described as pathogenic in human populations but that seem wild-type residues in non-human ortholog proteins, as well as intra-protein sites that significantly co-variate each other with two different algorithms, EV Mutation [66] and I-COMS [67].

The wide range of data output from MitImpact 3D was then enriched by HmtVAR [68], which hosts variability and pathogenicity data on human mtDNA variants, integrated with records retrieved from several online databases and in-house pathogenicity assessments, on the basis of various evaluation criteria. HmtVAR also presents manually curated tRNA variant attributes, but the most relevant resources dedicated to mitochondrial tRNAs that we explored were MITOTIP and PON-mt-tRNA.

MITOTIP mixed secondary structure information, structural analogies with other tRNA variants and conservation scores, providing the best prediction performances regarding specificity and sensitivity [69].

PON-mt-RNA, instead, is a posterior probability-based algorithm which computed a multifactorial score associating various features, such as sequence context and evidence of segregation, RNA secondary structure and tertiary interaction, functional assays, and evolutionary conservation [70].

### 2.8. Sub-Pathway Analysis

To emphasize the mitochondrial sub-pathways whose oxidative stress-induced alteration could be involved in retinal degenerations, the GO term enrichment analysis for mutated mtDNA genes was performed using the ClueGO (v. 2.5.7) (INSERM, Paris, France) [71] and CluePedia (v. 1.5.7) (INSERM, Paris, France) [72] plugins in Cytoscape (ver. 3.8.0) (National Institute of General Medical Sciences, Bethesda, MD, USA) [73]. ClueGO options have been set as follow: CLINVAR, GO (Biological Process, Cellular Component, Molecular Function and Immune System Process), INTERPRO, KEGG, REACTOME (Pathways and Reactions), WIKIPATHWAYS and CORUM 3.0 as selected ontologies; GO Tree Interval Min Level = 2 and Max Level = 9; GO Term/Pathway Selection Min # Genes = 2 and % Genes = 3.000; GO Term/Pathway Network Connectivity (Kappa Score) = 0.4; Statistics Options set on Enrichment/Depletion (Two-Sided hypergeometric test), with pV correction = Bonferroni step-down. CluePedia was used following default settings. Finally, only GO terms with *p* < 0.01 were selected.

## 3. Results

### 3.1. A2E Treatment Determined a Substantial Negative Effect on RPE Cells Survival

**Figure 3:**
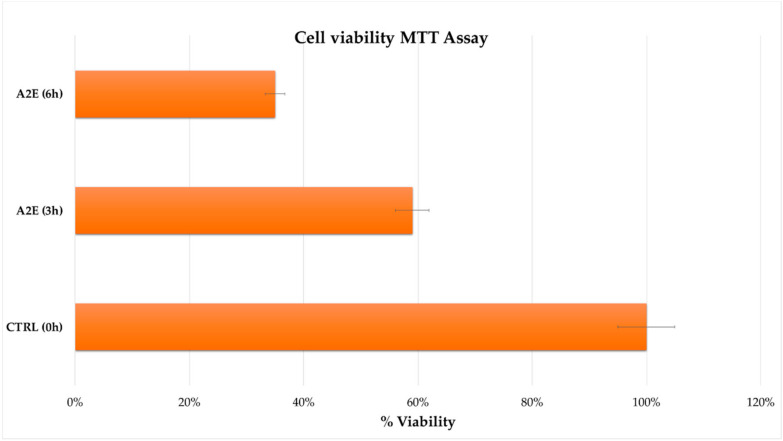
Cell viability from methylthiazolyldiphenyl-tetrazolium bromide (MTT) assay. Retinal pigment epithelium (RPE) cell survival percentage is expressed as mean of replicates ± standard error of mean, considering three replicates for each independent experiment (*n* = 3). Multiple *t*-tests were performed for statistical comparisons (*p* < 0.05). Results were assessed at both treatment considered time points (3 h and 6 h) compared to time zero untreated group.

The MTT assay highlighted a significant impact of A2E treatment on RPE cells viability in a time-dependent manner. In contrast to the control group, the viability of treated RPE cells was significantly decreased (*p* < 0.05), especially after 6 h from treatment (Figure 3).

### 3.2. Alignment and Assembly of mtDNAs

Once the quality of RNA (average RIN = 7.0) was assessed, the following sequencing globally generated about 100 million quality reads (mean mapping quality = 28), with a relevant number uniquely mapped to mtDNA, ranging from nearly two hundred reads generated from the 3 h treated sample (3h_RPE) to about 450,000 reads produced by Illumina paired-end experiment on untreated RPE cell transcriptome. Even if CLC Genomics Workbench and SMART2 were able to map mtDNA in all samples, the best efficiency was achieved by Trimitomics adapted workflow. It benefited from its ability to specifically work on RNA-Seq data and from the greater depth of initial raw data (not shown), highlighting elevated requirements requested by Bowtie2, NOVOPlasty, Trinity and Velvet algorithms. As is already known, gene expression profiling experiments that are looking for a quick snapshot of highly expressed genes may need 5–25 million reads per sample but, in order to achieve more detailed information such as alternative splicing, typically require 30–60 million reads per sample. Thus, such ranges reached by all samples ensured the good quality of the output. A detailed report of the alignment and assembly statistics is available in Table 1. Once produced, all partial or fully assembled mitogenomes were merged to obtain only one meta-mitogenome for each sample, needed for subsequent steps.

### 3.3. Mitogenome Annotations

**Figure 4:**
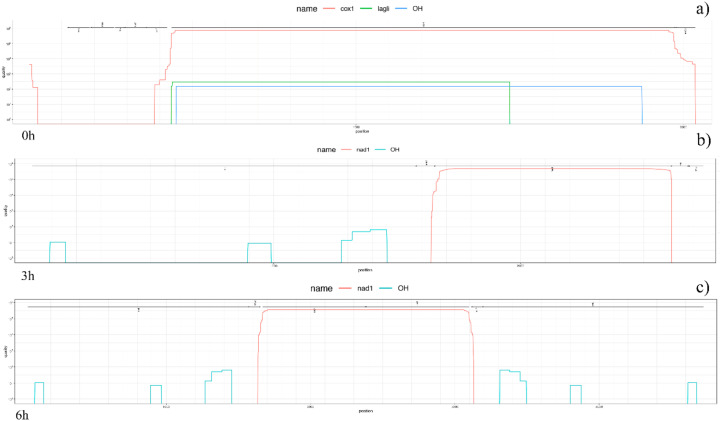
Protein coding gene plots. Figure shows annotated protein coding genes after congruences detection from BLASTX. The protein plot highlights the quality value (on a log scale) for each gene and each position if it is above the threshold. Different genes are evidenced by distinctive colors, with the initial hits corresponding to the represented “mountains”. The lines shown on the top represent the reported annotation. Annotation of protein coding genes reached the best results in untreated sample (**a**) while, in both time-related treated samples, the only protein coding gene identified with high quality was NAD1 (**b**,**c**).

**Figure 5:**
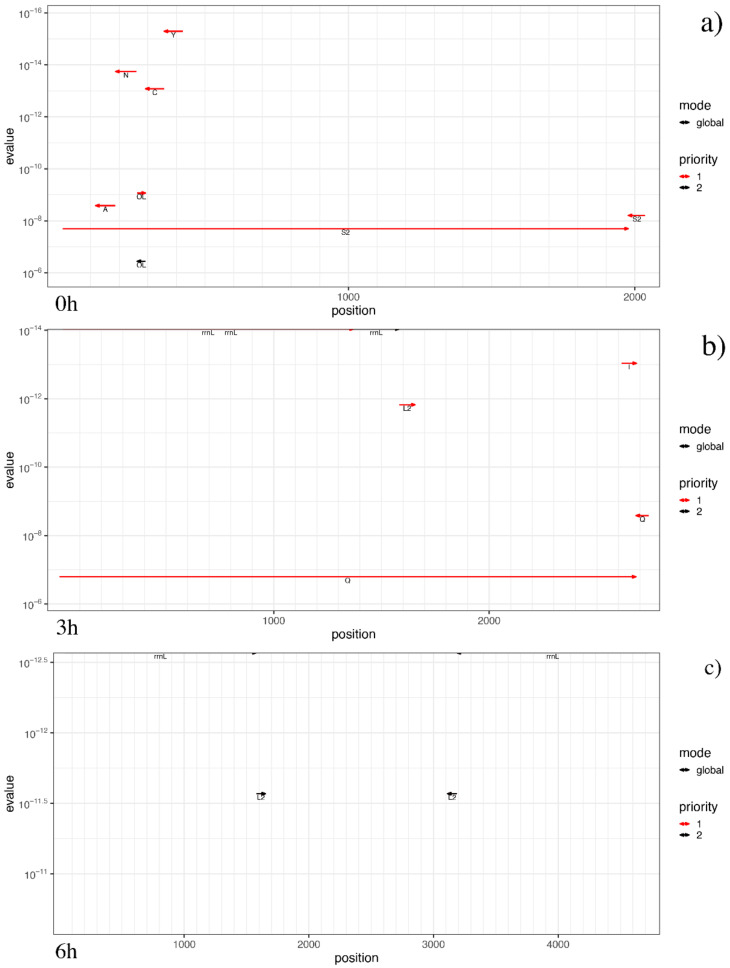
Non-coding RNA plots. The non-coding RNA plots show the hits from computation of rRNAs and tRNAs, differentiating between the hits from the global and local (if present) search by line type. The hits are prioritized: “1” refers to features set in the first round when tools take the prediction hit of each feature, while “2” refers to all other features set afterwards in the remaining unassigned regions. Reverse log scale for the e-value was considered. The untreated sample (**a**) showed the highest number of annotated tRNAs, as well as the widest positions covered among mitogenomes, while both treated samples (**b**,**c**) were the most reliable in rRNA.

**Figure 6:**
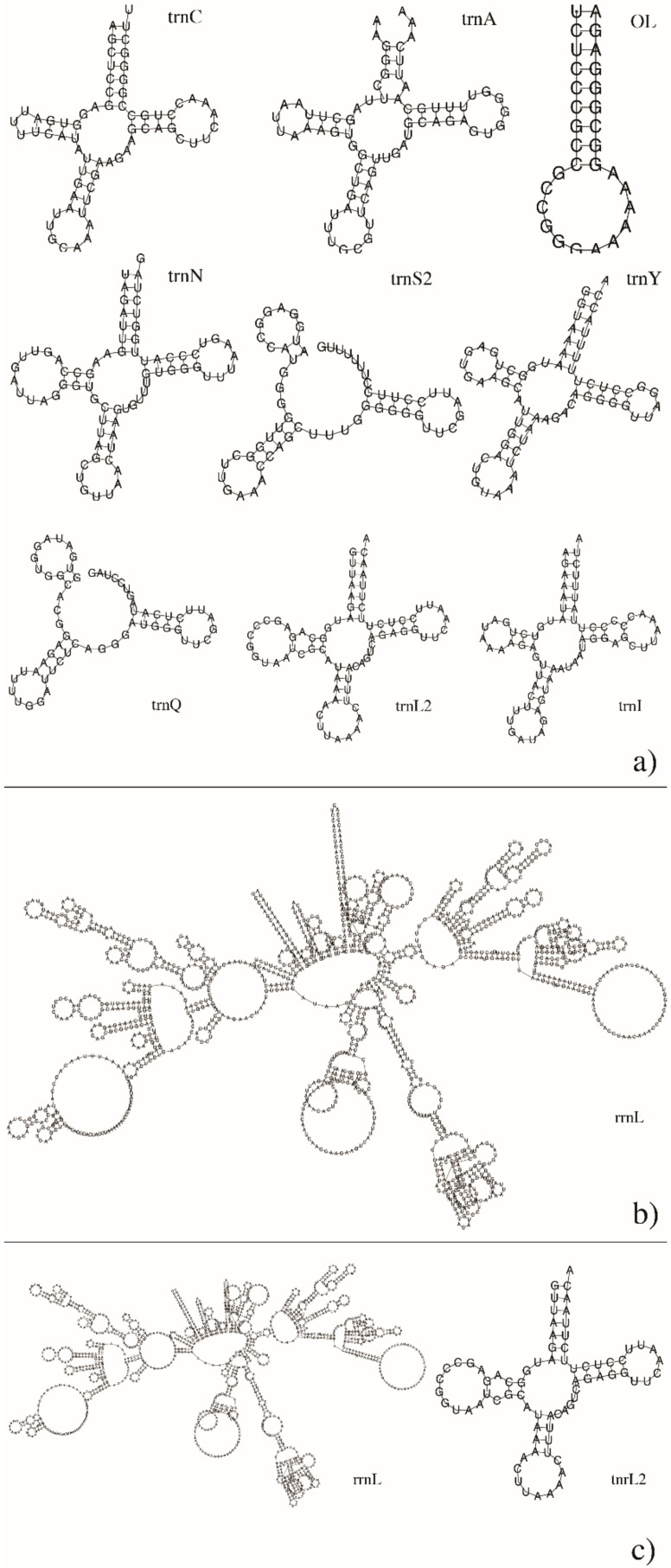
Non-coding RNA secondary structure computed by MITOS2. Figures represent main secondary structures of tRNAs ((**a**) and right element of (**c**)) and rRNAs ((**b**) and left element of (**c**)) output by MITOS2 analyses on all RPE cell transcriptomes.

The annotation of protein coding genes reached the best results in the untreated sample, in two genes with high quality score (~10^2^ for *LAGLI* and ~10^6^ for *COX1*) and the origin of heavy strand synthesis (OH) was detected. Curiously, in both time-related treated samples, the only protein coding gene identified with high quality was *NAD1*. The complete protein plots are shown in Figure 4. Furthermore, both treated samples were the most reliable in rRNA computation (e-values ~ 10^−14^–10^−12.5^), while the untreated one showed the highest number of annotated tRNAs, as well as the widest positions covered among mitogenomes. Interestingly, data annotation highlighted the longest tRNAs in both untreated and 3h_treated samples, even if with a good but not optimal significance (e-values ~ 10^−7^). Details on rRNA and tRNA annotations are shown in the non-coding plots of Figure 5. Intriguingly, we were also able to compute the secondary structure of several rRNAs and tRNAs for each sample, the most significant of which are represented in Figure 6. It is clear, especially for rRNAs, how changes in several nucleotides led to different RNA folding.

### 3.4. MtDNA Variant Calling and Annotations

**Figure 7:**
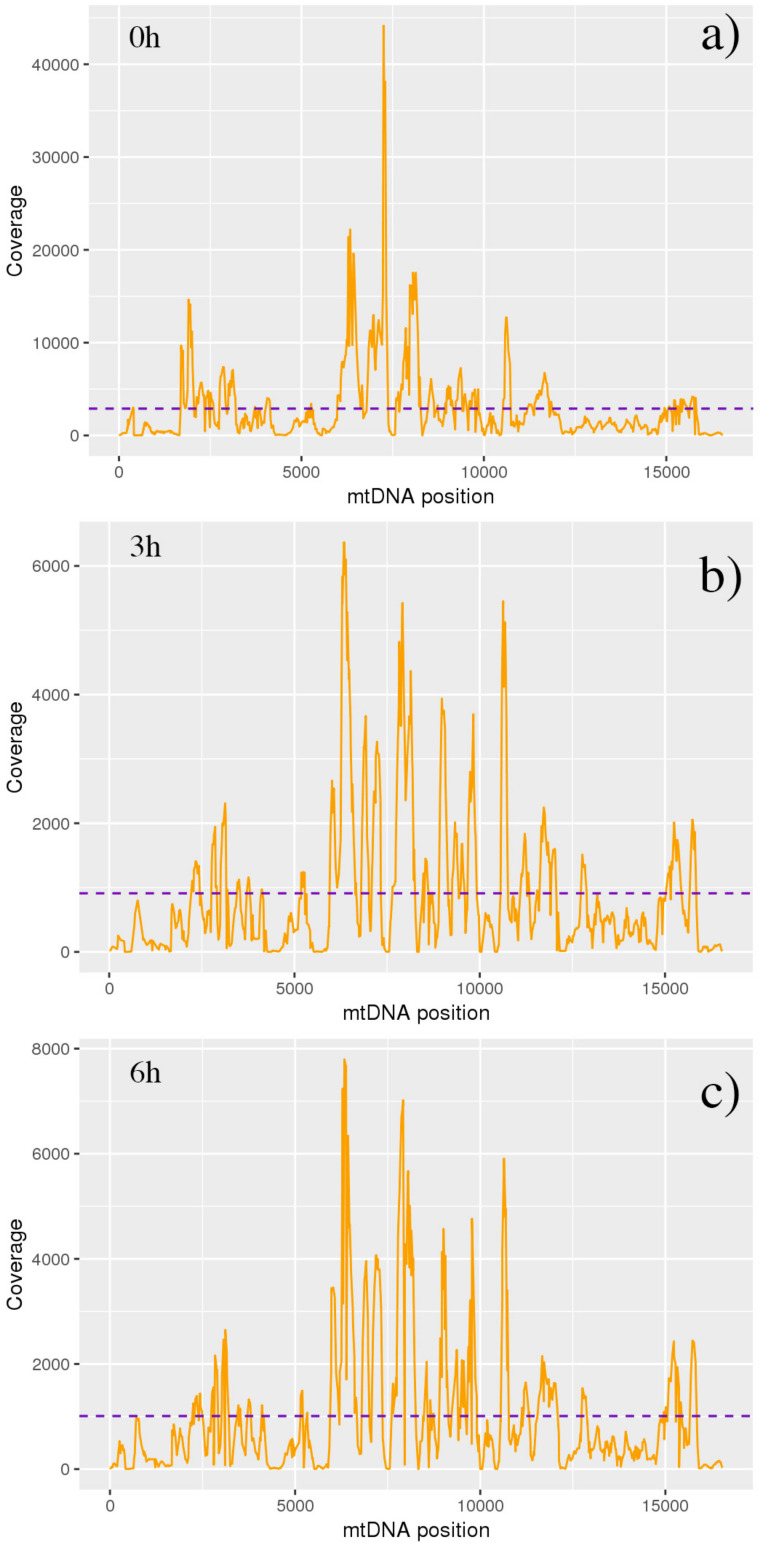
MtDNA-Server coverage plots. Figure shows coverage for each RNA-Seq sample, evaluated in all time-related conditions (**a** = 0 h, **b** = 3 h, **c** = 6 h). Coverage analysis permitted the detection of issues with incorrect concentration of polymerase chain reaction products for the used fragments. The highest coverage was reached around the 7500 nt position in untreated sample (**a**).

**Figure 8:**
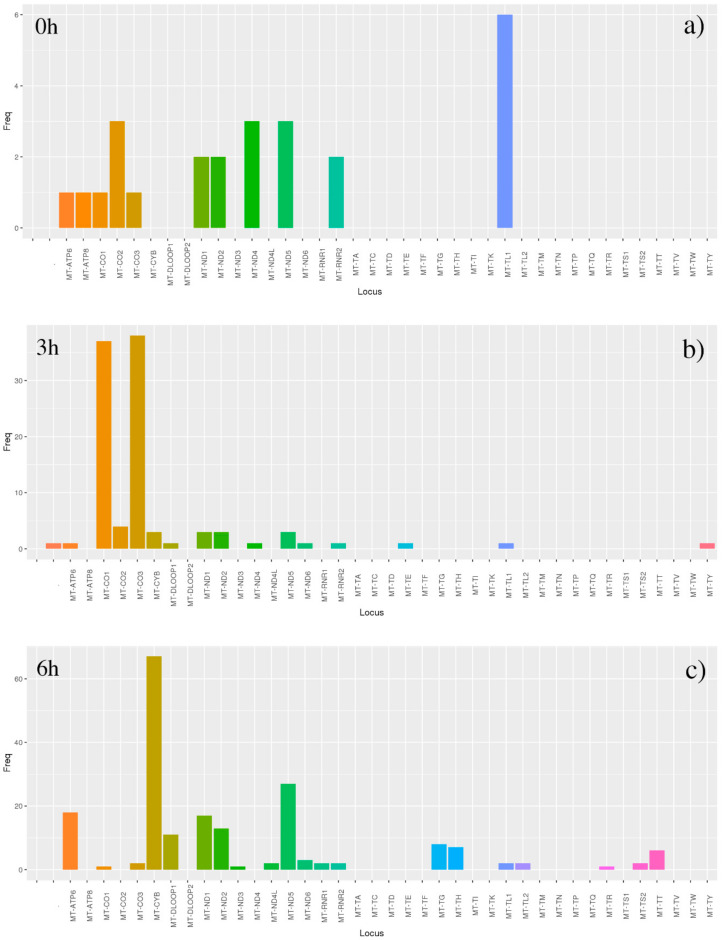
Results of heteroplasmic analyses by mtDNA-Server. The tool mtDNA-Server found heteroplasmic significant results in all samples, evaluated in all time-related conditions (**a** = 0 h, **b** = 3 h, **c** = 6 h), reaching the highest scores in 6 h treated sample. The highest peaks were reached by MT-CO1 and MT-CO3 loci in 3 h treated (**b**), and by MT-CYB locus in 6 h treatment (**c**).

The mtDNA-Server highlighted excellent results from previous alignment/assembly steps, reaching the highest coverage around the 7500 nt position in the untreated sample (Figure 7). Both time-dependent treated samples showed elevated level of heteroplasmic sites, with the highest peaks reached by *MT-CO1* and *MT-CO3* loci in 3 h treated, and by *MT-CYB* locus in 6 h treated (Figure 8).

RNA-seq analyses evidenced the highest number of heteroplasmic mtDNA variants in both treated samples (n_3h_ = 320, n_6h_ = 195), with the highest peak of unique annotated variants at 6 h (*n* = 45). Transitions were the most detected variants (only one T>G transversion was found), with an overrepresentation of A>G (*n* = 48) and C>T (*n* = 21) (Table S1). This result is in line with what is already known in literature [74]. VEP reached the highest number of annotated variants (n_0h_ = 23, n_3h_ = 24 and n_6h_ = 29), while MSeqDR, the most experimental data-rich database, identified just one more variant in the treated than the untreated group (n_0h_ = 20, n_3h_ = 21 and n_6h_ = 21), with 17 and 18 of them, respectively, in 3 h and 6 h treatment, also reported in the dbSNP database. These variants were prevalently in protein coding genes (*n*~20) with, respectively, 5 and 12 missense in 3 h and 6 h treated samples. Interestingly, the 6 h treatment resulted as the only showing a relevant variant in a rRNA coding gene. Regarding functional predicted consequences, four variants highlighted a possible pathogenic effect (m.11467 A>G; m.12372 G>A; m.14766 C>T; m.15326 A>G), while eight variants in untreated (m.73 A>G; m.150 C>T; m.263 A>G; m.456 C>T; m.2857 T>C; m.5656 A>G; m.16270 C>T; m.16304 T>G), in 3 h treated (m.73 A>G; m.150 C>T; m.263 A>G; m.750 A>G; m.951 G>A; m.5656 A>G; m.16270 C>T; m.16354 C>T) samples, and 13 (m.73 A>G; m.150 C>T; m.263 A>G; m.497 C>T; m.1438 A>G; m.3197 T>C; m.5656 A>G; m.12196 C>T; m.12308 A>G; m.16224 T>C; m.16270 C>T; m.16311 T>C; m.16519 T>C) and in the 6 h treatment one might exert a modifier role. Variant annotation summaries for all RNA-Seq samples are available in Table 2, while advanced details are available in Table S1.

### 3.5. In Silico Functional Consequences and Pathogenicity Predictions

The next step of the performed pipeline foresaw the use of in-silico predictions, to clarify the possible consequences of identified variants, especially ones which lack certain effects such as VUS.

Even if about 50 unique variants were detected by mvTool and MITIMPACT 3D throughout all samples, only about half of them were evaluated as damaging in investigated databases. In particular, the highest number of deleterious variants were identified by PROVEAN (18), Meta-SNP (20), PhD-SNP and SNAP (21), CADD and MToolBox (25), and Condel (28). Interestingly, 19 variants were computed by EVmutation, quantifying simultaneously the effects of multiple mutations by explicitly modeling interactions between all the pairs of residues in proteins (and bases in RNAs). The impact of the latter analysis was further corroborated by the total absence of CPD, which increased the probability that hypothesized effects of detected variants were truly positive. Curiously, HmtVAR classified most of the identified variants as coding sequence (29) and regulatory (11), providing important evidence on the different kinds of impact probably determined by these variants. Furthermore, pathogenic or likely pathogenic variants were detected in both treated samples, and two tRNA variants found in 6 h treated samples were also found in the Pon-mt-tRNA database. The scenario could also represent an important finding as the most of variants seem to be new mutations, as appeared from both dbSNP and CLINVAR (Table 3).

Regarding haplogroup classification, RPE cell transcriptomes showed a putative assignment to U5b1b1+@16192 haplogroup, as outputted by the mtDNA-Server.

### 3.6. Sub-Pathways Analysis of mtDNA Mutated Genes Suggested a Positive Regulation of ATP Metabolism

**Figure 9:**
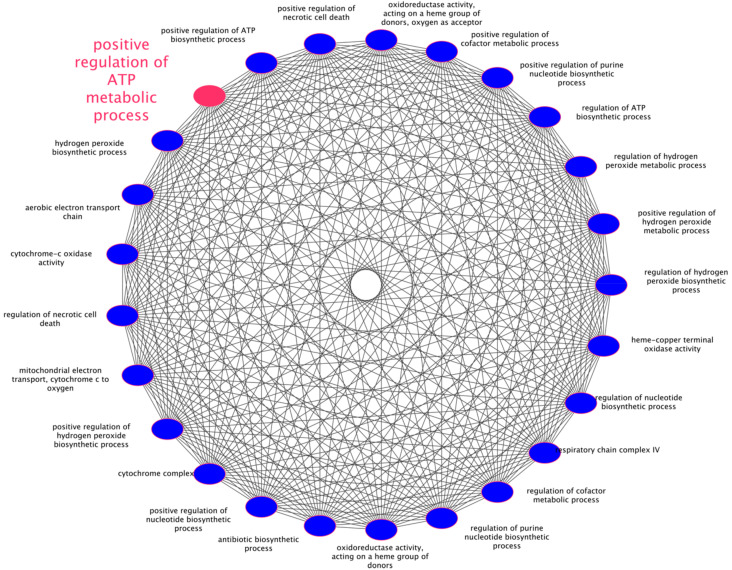
Pathway analysis by Cytoscape and its plugins. The spherical graph represents the significant pathways computed by Cytoscape, ClueGO and CluePedia. It is clear that regulation of adenosine triphosphate (ATP) biosynthesis represents the most altered pathway following the oxidative treatment with A2E.

All the 25 clustered sub-pathways obtained from enrichment of mtDNA mutated genes, derived from transcript analysis, thanks to Cytoscape and its plugins ClueGO and CluePedia gave statistically significant results (Figure 9). Among them, seven pathways showed the highest probability of association (Bonferroni step-down corrected *p* < 0.001), highlighting the most altered functions of mitochondria of RPE stressed cells: “Positive regulation of hydrogen peroxide metabolic process” (*p* = 1.45 × 10^−3^), “Regulation of hydrogen peroxide biosynthetic process” (*p* = 1.80 × 10^−3^), “positive regulation of hydrogen peroxide biosynthetic process” (*p* = 1.03 × 10^−3^), “positive regulation of necrotic cell death” (*p* = 1.80 × 10^−3^), “regulation of nucleotide biosynthetic process” (*p* = 1.55 × 10^−3^), “positive regulation of cofactor metabolic process” (*p* = 1.80 × 10^−3^) and “positive regulation of ATP metabolic process”, that represented the hub-pathway with the highest significance (*p* = 8.10 × 10^−11^). Further details on significant pathways are available in Table S2.

## 4. Discussion

The advent of “omics” sciences and the development of specific bioinformatics algorithms gave systematics to analyses involving mtDNA and its linkage to related diseases [75]. Among disorders already known to be determined by mtDNA variants [76], the best characterized are oxidative stress induced ones, such as Alzheimer’s, Parkinson’s and other neurodegenerative disorders [77].

One of the not totally explored field of neurodegenerative pathologies associated to mtDNA impairments regards the inherited retinal dystrophies (IRDs) [78,79,80]. The only available data are related to Leber Hereditary Optic Neuropathy (LHON) and to age-related degenerations, like AMD [81], even if clear molecular mechanisms linking mtDNA alterations to these pathologies are already not clear.

In order to shed light on such molecular aspects, we evaluated the possible mutagenic effects of blue light photoactivated A2E on RPE cells in a time-dependent experiment. We analyzed mitochondrial transcripts and, with an innovative bioinformatic pipeline, we derived mtDNA information, permitting a comparison of the possible mtDNA damage after 3 and 6 h from treatment versus untreated cells.

It is well known that blue-violet light (415–455 nm) within the solar spectrum exerts a toxic effect to the retina, generating the highest amount of ROS and the highest level of mitochondrial dysfunction [82]. Moreover, the exposure of cells to A2E determines an increased production of ROS and NOS, determining a mitochondria-related oxidative stress [83].

Such effects were confirmed by our A2E treatment, showing a huge decrease in cell viability within the time range of six hours. The most challenging aspect of this scenario is represented by molecular mechanisms able to induce such mitochondrial impairments. We hypothesized that A2E might act as a mutagenic compound towards mtDNA, as supposed by the increased number of variants that emerged after 3 h and 6 h from treatment compared to untreated RPE cells. This hypothesis is supported by intrinsic characteristics of both interacting protagonists, mtDNA and A2E. The mtDNA is greatly susceptible to damage, due to the absence of histones, localization within the matrix, high rate of transcription with the lack of intron and DNA damage response (DDR), less efficient than in the nucleus [84,85]. The A2E presents an aldehydic component within its chemical structure, which can cross-link with different chemical moieties on DNA, forming adducts to deoxyguanosine, deoxyadenosine and deoxycytidine, leading to many toxic consequences such as mutagenesis [85,86,87].

The mitochondrial assembly pipeline was able to compute entirely both *cox1* and *lagli* encoding genes only in untreated cells, while in both time-related treated RPE cells only the *nad1* gene (absent in untreated samples). A2E can act as proapoptotic molecule, specifically targeting cytochrome oxidase (COX) and inhibiting oxygen consumption synergistically with light [88], probably explaining the lack of full *cox1* sequence, and the wealth of variants in computed portion, as observed after 3 h of treatment. The *lagli*, instead, is a maturase domain of enzymes promoting RNA splicing and intron homing [89], two fundamental cellular activities probably impaired during treatment, denoting the progress of apoptotic process. The detection of the *nad1* gene sequence in both 3 h and 6 h treated samples could reflect the abundance of mutant forms of NAD^+^, following the oxidative stress condition of RPE cells. Even if the de novo pathway of NAD^+^ biosynthesis seems to be unaffected in retinal aging and various conditions of retinal degenerations, the salvage pathway was found to be significantly altered, determining a significant decline in oxidative phosphorylation and an up-regulation of glycolytic respiration [90,91]. The final effect of these impairments is the mitochondrial ATP production decrease in the presence of A2E [92].

Differences in the assembly of mtDNA between untreated and treated RPE cells were also evidenced in 16S (rrnL) and tRNA genes. Mitochondrially encoded 16S rRNA is required for the biosynthesis of mitochondrial-derived peptide humanin, exerting cytoprotective and neuroprotective activities [85]. Thus, its detection in both time-dependent treated samples could represent a final attempt to avoid the advancing cell death, also represented by the almost total absence of tRNAs (in the 6 h treated sample only the L2 connector of tRNAs was computed).

As already cited, the effects of A2E on RPE are also time-dependent, and its accumulation exacerbates the effects of mitochondrial dysfunction [93]. Cells do not die immediately but suffer genotoxic damages with the formation and accumulation of mutagenic DNA lesions (double strand breaks and FPG sensitive lesions) [94,95,96]. Failure in repair of damaged mtDNA may turn into mutation, which can be maternally inherited. For example, if base excision repair (BER) fails in removing 8-oxoG, a major oxidative modification of mtDNA, this one can be further oxidized to produce its more stable and mutagenic forms, which may interfere with mtDNA replication [84].

As we demonstrated, it is very likely that the damage induced in mtDNA will be localized in a coding sequence corresponding with either the polypeptide-encoding genes or the RNA, thereby limiting the biosynthesis of fully functional proteins [8]. Generally, mtDNA mutations determine a clinically observable phenotype only if the proportion of mutant mtDNA exceeds a high threshold value, often 80–90%. Most of homoplasmic mtDNA mutations are neutral polymorphisms, but several of them or combinations of them in mtDNA haplogroups could be associated with the onset or progression of retinal diseases [8]. Nevertheless, most mtDNA mutation-related pathologies are heteroplasmic, with heteroplasmic subjects with high percentage of mutant mtDNA manifesting the increasing risk of vision loss [97].

Untreated samples showed the highest frequency of heteroplasmic variants in *MT-TL1*, encoding for the tRNA^leucine1^, belonging to no protein complexes, but known to present the most frequent mutations which cause many mitochondria-related diseases, except retinal ones [98]. After treatment, the damage sites reached all oxidative phosphorylation system (OXPHOS) complexes. The complex IV of cytochrome c oxidase evidenced the highest heteroplasmic variant frequency in *MT-CO1* and *MT-CO3* genes after 3 h from treatment. Subsequently, at the end of the experiment (6 h), all the other complexes presented the highest rate of heteroplasmic variants in several protein encoding genes. In detail, they were the NADH dehydrogenase subunit 5 (*MT-ND5*) encoding gene belonging to the first complex, the cytochrome b (*MT-CYB*) encoding gene involved in the third complex, and the ATP synthetase subunit 6 (*MT-ATP6*) constituting a part of the fifth complex.

As evidenced by pathway analysis, mutated mtDNA genes play a pivotal role in the positive regulation of ATP metabolic process, suggesting a huge impairment in the energetic balance of the cell. It is very probable that mtDNA damage affects genes encoding the mitochondrial electron transport chain at various levels, resulting in ROS overproduction, which leads to more damage to mtDNA. Such injuries, as already cited, could be worsened by A2E or other radical species such as hydrogen peroxide, whose biosynthesis resulted from pathway analysis, and whose role was already linked to decreased redox function in RPE cells [84]. Thus, when antioxidants enzymes or DNA repair systems fail, accumulated damage to mtDNA can result in mitochondrial dysfunctions, energy crisis, cell degeneration and death, as observed in many retinal diseases [8].

## 5. Conclusions

The continuously updated next generation sequencing technologies allowed researchers to access to the entire mtDNA sequencing data, which is constantly being increased. We used, for the first time, an integrated approach starting from RNA rather from DNA, trying to maximize the innovation of our work.

However, sequencing the whole cell transcriptome instead of mtDNA could determine several limitations: (1) RNA-Seq would result in a much lower coverage with the same amount of sequencing mtDNA data; (2) it is more difficult to cover the full-length mtDNA, including all regulatory regions; (3) deletions and rearrangements could be lost from the transcriptomics data.

Furthermore, there are several critical restrictions that should be evaluated during mtDNA damage analysis interpretation, such as DNA fragmentation, greater diversity in both the number of mitochondria per cell as well as the number of mtDNA copies per mitochondria, mitochondrial haplogroups, identification of helper or synergistic mutations and co-occurrences of variants.

Taking into account such critical issues, we highlighted how a high oxidant environment can alter the physiological activities of mitochondria, acting on its own mtDNA. Thus, trying to clarify how a frequently retinal produced compound such as A2E could exert mutagenic effects on RPE mtDNA, we hypothesized new scenarios that might link unknown molecular aspects of the cell to the onset/progress of eye-related neurodegenerative pathologies.

These powerful results, along with further pathway analyses to decrypt the biological role of the involved genes, should be incorporated into clinical diagnostic settings, as they are hypothesized to modulate the phenotypic expression of mtDNA pathogenic variants. In this way, an integrative analysis of mitochondrial genome, together with the nuclear genome, could drastically improve the field of precision molecular medicine, with the final goal being to improve patients’ healthcare.

## Figures and Tables

**Table 1:** Alignment and assembly statistics of RNA-Seq analyzed samples. Table shows main features related to mitochondrial transcripts shared in overall RNA, as well as mapping and assembly steps performed on RNA-Seq samples by CLC Genomics Workbench, SMART2 and TRIMITOMICS algorithms. Values are reported as the mean of the three algorithms and of all three biological replicates for each sample.

Statistics Feature	0h_RPE	3h_RPE	6h_RPE
**Overall Passed Reads**	52,752,353	26,262,324	19,520,749
**Passed FWD Reads**	51,913,969	16,056,486	19,301,263
**Passed REV Reads**	838,384	205,838	219,486
**Mapping Quality OK**	440,014	132,054	162,126
**Mapping Quality BAD**	29,372	10,603	15,517
**Unmapped Reads**	0	0	0
**Base Read Quality OK**	52,752,353	16,262,324	19,520,749
**Base Read Quality BAD**	15,127,187	3,805,671	4,905,387
**Bad Alignment**	0	0	0
**Duplicates**	0	0	0
**Short Reads (<25 bp)**	0	0	0
**Mitochondrial Transcripts**	29,123	27,095	25,471

**Table 2:** RNA-Seq sample variant annotation summary. Table reports a summary of detected variants, together with key mitogenome variant annotations from examined databases by mvTool, MitoBreak and MitoMaster.

			**0h_RPE**	**3h_RPE**	**6h_RPE**
	Heteroplasmic Variants (Global)		26	320	195
	Unique annotated variants (Global)		34	29	45
**mvTool**	MSeqDR Community Data Population	N° Variants	20	21	21
		Mitomap Disease	4	4	4
		Mitomap Status	3 Reported, 1 Conflicting	3 Reported, 1 Conflicting	3 Reported, 1 Conflicting
		HmtDB Pathogenicity	8 Pending, 4 Benign	8 Pending, 4 Benign	9 Pending, 4 Benign
	Disease and Phenotypes	dbSNP	17	17	18
		MSeqDR Clinical Significance	4 Likely Pathogenic, 2 Not Provided	4 Likely Pathogenic, 2 Not Provided	4 Likely Pathogenic, 2 Not Provided
		HmtDB Disease	4	4	4
		COSMIC	1	1	1
		ICGC	2	2	2
	VEP	Impact	11 Low, 4 Moderate, 8 Modifier	11 Low, 5 Moderate, 8 Modifier	11 Low, 4 Moderate, 13 Modifier
		Biotype	23 Protein Coding	24 Protein Coding	28 Protein Coding, 1 mt_rRNA
		Consequence Terms	16 Synonymous, 3 Missense, 4 Upstream	14 Synonymous, 5 Missense, 4 Upstream	11 Synonymous, 12 Missense, 4 Upstream, 1 Non-coding Transcript Exon
	CADD	Raw Raknscore	1	1	1
	HmtDB Patho Table	N° Variants	3	3	3
		Pathogenicity	1	1	1
**MitoBreak**		N° Deletions	0	1	0
		Healthy Tissue	0	Aged Tissues	0
		Del of replication origins	0	None	0
		Location of the deleted regions	0	Inside the major arc	0
**MitoMaster**		N° Variants	20 Transitions	20 Transitions, 1 Transversion	21 Transitions
		Mut Type	15 Coding, 5 Non-coding	17 Coding, 4 Non-coding	16 Coding, 5 Non-coding
		Patient Report	4	4	4

**Table 3:** In-silico functional prediction analyses report. Table shows principal results on pathogenicity prediction of identified variants through all samples. The main macro-resources used are highlighted in red.

**MitImpact 3D**		**0h_RPE**	**3h_RPE**	**6h_RPE**	**MitImpact 3D**		**0h_RPE**	**3h_RPE**	**6h_RPE**	**MitImpact 3D**		**0h_RPE**	**3h_RPE**	**6h_RPE**
**N° Variants**		34	29	45	**N° Variants**		34	29	45	**N° Variants**		34	29	45
**PolyPhen 2**	Benign	9	7	8	**FatHmm**	Neutral	9	8	10	**Mutation Assessor**	Neutral Impact	5	3	4
	Probably damaging	5	5	8		Deleterious	5	4	6		Low impact	3	2	4
**SIFT**	Neutral	12	9	13	**PROVEAN**	Neutral	9	8	10		Medium impact	2	2	4
	Deleterious	2	3	3		Deleterious	5	4	9		High impact	3	3	3
**EFIN_SP**	Neutral	12	10	13	**CADD**	Neutral	7	4	6	**PhD-SNP**	Neutral	7	4	10
	Damaging	2	2	3		Deleterious	7	8	10		Disease	7	8	6
**EFIN_HD**	Neutral	11	9	12	**PANTHER**	Neutral	10	7	10	**SNAP**	Neutral	8	5	8
	Damaging	3	3	4		Disease	4	3	6		Disease	6	7	8
**Meta-SNP**	Neutral	8	6	8	**Condel**	Neutral	4	4	6	**MToolBox**	Neutral	6	5	6
	Disease	6	6	8		Deleterious	10	8	10		Deleterious	8	7	10
**CAROL**	Neutral	10	8	10	**COVEC_WMV**	Neutral	10	8	10	**APOGEE**	N	11	10	11
	Deleterious	4	4	6		Deleterious	4	4	6		P	3	2	5
**Mutation Taster**	Polymorphism	10	9	13	**CLINVAR**	Yes	2	2	2	**Mitoclass1**	Neutral	10	7	9
	Disease Causing	2	3	2		No	12	10	14		Damaging	4	5	7
**dbSNP**	SNP	4	4	6	**Mitomap**	Yes	0	0	2	**DDG_Intra**	Yes	8	6	8
	New Mutation	10	8	10		No	14	12	14		No	6	6	8
**DDG_Inter**	Yes	2	1	1	**EV_Mutation**	Yes	8	5	10	**Pon-mt-tRNA**	N° Variants	0	0	2
	No	12	11	17		No	6	7	6		Neutral or Likely Neutral	/	/	2
**HmtVAR**	N° Variants	13	19	14	**HmtVAR**	N° Variants	13	19	14	**HmtVAR**	N° Variants	13	19	14
	CDS	7	14	8		rRNA	3	1	1		Polymorphic	2	2	0
	Regulatory	3	4	4		tRNA	0	0	2		Likely Polymorphic	1	0	1
	Likely Pathogenic	1	0	2		Pathogenic	0	1	0		Unavailable	9	16	12

## Supplementary Materials

The following are available online at https://www.mdpi.com/2076-3921/9/11/1158/s1, Table S1: MtDNA Variant Calling and Annotations details; Table S2: Details of Sub-Pathways Analysis of mtDNA Mutated Genes.

## Data Deposition

According to the journal guidelines, RNA-Seq data was deposited in NCBI’s Sequence Read Archive (SRA) and assigned to the project identifier PRJNA622997.
